# Recurrence quantification analysis for fine-scale characterisation of arrhythmic patterns in cardiac tissue

**DOI:** 10.1038/s41598-023-38256-w

**Published:** 2023-07-22

**Authors:** Radek Halfar, Brodie A. J. Lawson, Rodrigo Weber dos Santos, Kevin Burrage

**Affiliations:** 1 IT4Innovations, VSB - Technical University of Ostrava, 708 00 Ostrava, Czech Republic; 2 ARC Centre of Excellence for Plant Success in Nature and Agriculture, Queensland University of Technology, Brisbane, 4000 Australia; 3 Centre for Data Science, Queensland Univeristy of Technology, Brisbane, 4000 Australia; 4 Graduate Program in Computational Modeling, Universidade Federal de Juiz de Fora, Juiz de Fora, 36036-330 Brazil; 5 Department of Computer Science, University of Oxford, Oxford, UK

**Keywords:** Applied mathematics, Computational science

## Abstract

This paper uses recurrence quantification analysis (RQA) combined with entropy measures and organization indices to characterize arrhythmic patterns and dynamics in computer simulations of cardiac tissue. We performed different simulations of cardiac tissues of sizes comparable to the human heart atrium. In these simulations, we observed four classic arrhythmic patterns: a spiral wave anchored to a highly fibrotic region resulting in sustained re-entry, a meandering spiral wave, fibrillation, and a spiral wave anchored to a scar region that breaks up into wavelets away from the main rotor. A detailed analysis revealed that, within the same simulation, maps of RQA metrics could differentiate regions with regular AP propagation from ones with chaotic activity. In particular, the combination of two RQA metrics, the length of the longest diagonal string of recurrence points and the mean length of diagonal lines, was able to identify the location of rotor tips, which are the active elements that maintain spiral waves and fibrillation. By proposing low-dimensional models based on the mean value and spatial correlation of metrics calculated from membrane potential time series, we identify RQA-based metrics that successfully separate the four different types of cardiac arrhythmia into distinct regions of the feature space, and thus might be used for automatic classification, in particular distinguishing between fibrillation driven by self-sustaining chaos and that created by a persistent rotor and wavebreak. We also discuss the practical applicability of such an approach.

## Introduction

Cardiac arrhythmias are a prevalent disease worldwide and a leading cause of death. The onset of these arrhythmias is complex and remains difficult to anticipate. In 1952, Hodgin and Huxley presented the first mathematical model of cell action potential (AP)^1^, which is a critical component of furthering our understanding of arrhythmia in a mechanistic way^2,3^. Some more modern examples include the pairing of simulations of mathematical models of cardiac activity with data to inform the study, and treatment, of atrial flutter^4,5^ and atrial fibrillation^5–7^.

A particular topic of interest is ablative surgery, in which small scars on the heart muscle are created, blocking off regions thought to be producing or sustaining arrhythmogenic dynamics^4,8^. Given this treatment’s inconsistent success rate and invasive nature, there has been much interest in learning how to identify regions for ablation. One common idea is to target complex fractionated atrial electrograms (CFAEs), especially as a supplementary treatment if the pulmonary veins (a common trigger zone for atrial fibrillation) have already been electrically isolated^9^. Precise definitions of CFAEs vary, but the goal is to identify regions of conduction slowing/block, anchor points for re-entrant activation, sites of wavebreak or other pro-arrhythmic phenomena from electrograms. The issue is that simple criteria for identifying CFAEs are also prone to incorrectly labelling large regions of tissue not critical for atrial fibrillation maintenance as dangerous^10^.

A natural path forward, then, is to improve the identification of ablation targets via more sophisticated analysis of electrograms. In this work, we use recurrence quantification analysis (RQA) combined with entropy measures and organization indices to characterize arrhythmic patterns in computer simulations of cardiac tissue. RQA has emerged as a tool with great potential in electrophysiology, owing to its established history in capturing the regular movements of nonlinear dynamical systems, identifying transitions between regular and chaotic states, and enabling the study of unstable periodic orbits^11,12^. In the past, RQA has been used to analyze the relationship between atrial rate and spectral centre frequency^13^, to quantitatively analyse CFAEs^14,15^, to classify atrial electrograms as normal, fractionated or temporally unstable^16–18^, and to examine dynamics before and after ablative treatment^19^. This is possible even for short electrogram sequences^17,20^.

Most similar to our study, RQA has been used, together with sample entropy, to distinguish between areas with or without rotors^21^, by applying RQA to bipolar electrogram readings that can identify wavefront direction. Sites behaving actively or passively in the context of fibrillation have been identified using 15 s^22^ or 5 s^23^ signals. In this work, we examine a variety of RQA and related metrics applied to much shorter (1 s) AP traces. These traces form cardiac activation maps, which are an essential tool for the treatment of cardiac arrhythmias^24^. Furthermore, the method’s advantages for finding residual conduction after ablation of the mitral isthmus and thus ensuring its complete blockage are shown^25^ and thanks to the constant technological development of this method, new possibilities for its use are constantly being found^26^. We demonstrate that by appropriately combining RQA metrics, along with their spatial correlation, we can identify the changing nature of electrical signalling. In particular, we show how samples of single-site AP traces can be monitored for the presence of a stable rotor. We also demonstrate the use of RQA-based features to differentiate regular activation due to spiral waves (including separation of anchored and meandering spirals) and fibrillation (including separation of self-sustained and rotor-driven fibrillation) when high-resolution spatial data is available. These techniques also could serve as powerful tools for automated analysis of *in silico* simulation data, where full spatial AP data is available.

## Materials and methods

### Electrophysiological scenario simulation

The propagation of the action potential (AP) in cardiac tissue is modeled using the monodomain equation in terms of a dimensionless transmembrane potential, *u*,

1 $$\begin{aligned} \frac{\partial u}{\partial t} = D \left( \frac{\partial ^2 u}{\partial x^2} + \frac{\partial ^2 u}{\partial y^2} \right) - J_{\text {ion}} - J_{\text {stim}}. \end{aligned}$$

Membrane potential diffuses with isotropic conductivity *D*, representing the flow of ions between myocytes through their gap junctions. Membrane potential also changes with the flow of ions in and out of cells, $J_{ion}$, and any externally provided stimulus current, $J_{stim}$. Here, $J_{ion}$ is represented by the model of Fenton and Karma^27^. This is a phenomenological model that simplifies ion transport to single currents representing the fast inward, slow inward, and slow outward flow of positive ions through the cell membrane ($J_{fi}$, $J_{si}$ and $J_{so}$, respectively),

$$\begin{aligned} J_{\text {ion}}=-J_{\text {fi}}-J_{\text {so}}-J_{\text {si}}. \end{aligned}$$

Further information on the definition of this model, including parameter values used, can be found in the Supplementary material (Table S1).

We consider two different scenarios relevant to cardiac arrhythmia. In the first scenario, we simulate a two-dimensional slice of tissue of dimension $12.5\times {12.5}\,{\text{cm}}$, this large size is chosen to allow single simulations to contain multiple dynamical behaviours in accordance with other similar studies^23^. No-flux boundary conditions are applied to domain edges. To produce the type of chaotic dynamics observed during fibrillation, we provide a rapid pacing stimulus, with a period of 31 ms. Numerical discretisation parameters for this scenario are grid spacing $\Delta _x = {0.25}\,{\text{mm}}$ and timestep $\Delta _t = {0.1}\,{\text{ms}}$.

In the second scenario, two-dimensional tissue slices of dimension $4\times {4}\,{\text{cm}}$ are used, a size corresponding to the heart atrium. In this scenario, scar regions are added to make the tissue heterogeneous, creating the potential for spiral waves to be stabilised by the obstructed region^28^. The scar is created by placing two concentric circles, with a fixed probability of cell damage for sites within the inner circle and a linear decrease in probability to zero along the radius of the outer ring. This creates a border zone, known to be an important component of a scar regions’ arrhythmogenic impact^29^, as well as being a strong predictor of post-infarct mortality^30^. Damaged tissue is modelled as non-conductive (applying no-flux boundary conditions to boundaries between damaged and healthy tissue). In order to generate many different types of activation dynamics, a wide range of magnitudes of tissue damage are simulated, from no damage to complete destruction of tissue in the scar region. This scenario used $\Delta _x = {0.2}\,{\text{mm}}$ and $\Delta _t = {0.05}\,{\text{ms}}$. In the simulations, the diffusion was $D_{xx} = D_{yy} = {0.25}\,{\text{cm}}^{2}/{\text{s}}$. If there was a scar in a given site, the diffusion coefficient was set to $D={0}\,{\text{cm}}^{2}/{\text{s}}$.

### Signal analysis

Analysis of membrane potential time series is here approached using RQA^11,31^, an important method for nonlinear data analysis. RQA is based on the analysis of recurrence in a dynamic system (events of a dynamic system returning to a previously visited area in the phase space), as summarised by the recurrence plot (RP). The RP is a two-dimensional array of the values zero and one, with a value of one in position (*i*, *j*) corresponding to the system being in the same place in phase space at times $t_i$ and $t_j$. That is, the non-zero elements in the array mark events of recurrence.

The RP is calculated using the equation

2 $$\begin{aligned} R_{i,j}=\theta (\epsilon -||{\textbf{x}}_i-{\textbf{x}}_j||) \text {, ~~~ }{\textbf{x}}_i \in \mathbb {R}^m \text {, ~~~ }i,j=1\dots N. \end{aligned}$$

Here $\textbf{x}_i$ denotes the values of the system’s *m* dependent variables at the moment of its *i*-th snapshot, with *N* the total number of snapshots. The Heaviside function $\theta (x)$,

3 $$\begin{aligned} {\displaystyle \theta (x):={{\left\{ \begin{array}{ll}1,&{}x>0\\ 0,&{}x\le 0\end{array}\right. }}}, \end{aligned}$$

specifies that recurrence occurs when the difference falls under the threshold $\epsilon$ for some choice of norm $\Vert \cdot \Vert$. Here, we use the Euclidean norm. Once the RP is obtained, a number of different informative quantities can be calculated from the arrangement of its zero and non-zero elements. Many of these quantities consider the occurrence of diagonal strings of non-zeroes in the RP, as these correspond to system dynamics recurring throughout a window of time, instead of just coinciding at a single moment. In this paper, the following RQA measures will be used:

**Table:** 

**RQA measure**	**Description**
*REC*	Percentage of recurrence points in a recurrence plot
*DET*	Percentage of recurrence points that form diagonal lines
*RATIO*	Ratio between *DET* and *REC*
$L_{max}$	Length of the longest diagonal string of recurrence points
$L_{mean}$	Mean length of the diagonal lines
*DIV*	The inverse of $L_{max}$
*ENTR*	The Shannon entropy of the diagonal line lengths distribution
*LAM*	Percentage of recurrence points that form vertical lines
$V_{max}$	Length of the longest vertical line
$V_{mean}$	Mean length of vertical lines

In addition to these RQA metrics, we also consider the organization indices (OI) for these time series. Widely used in cardiac electrophysiology studies^32–39^, organization indices are a measure of order (or disorder) in a time series, calculated using its representation in the frequency domain. Specifically, the *n*th organization index is defined as the proportion of power in the power spectrum that is contained within the *n* highest-power peaks. A high organization index, with large proportions of the power in the power spectrum concentrated in a small number of peaks, corresponds to regular activation of a location at some fixed frequency(ies). Calculations of the power contained within a peak will sometimes also include the power associated with its harmonic frequencies, but here these are not included.

Finally, we analyze time series in terms of their entropy. Accurate entropy calculation requires large amounts of data and is very sensitive to the noise of the system under investigation, and so is not suitable here. To overcome this limitation, Pincus *et al.*^40^ developed an approximate entropy measure (*ApEnt*), based on searching for similar subsequences in the analyzed time series. In order to avoid the occurrence of natural logarithms of zero in the *ApEnt* calculation, each sequence is counted as similar to itself, although this introduces a bias^41^. Richman and Moorman^41^ proposed sample entropy (*SampEnt*) as a modified entropy measure that is independent of the length of the data, is bias-free, and also requires fewer operations to calculate^42^. Further information about the calculation and use of these entropy metrics is offered by Delgado-Bonal and Marshak^43^. We consider both *ApEnt* and *SamptEnt* in this work.

All metrics are calculated from time series of length 1000 ms, with a sampling frequency of 1000 Hz.

### Spatial analysis

As a means of automatically identifying different types of arrhythmic dynamics, we take together the values of the time series metrics discussed in the previous section at different spatial locations. The approach is based on the intuition that overall measures of order/disorder in the tissue distinguish between tachycardic and fibrillatory patterns of activation, and the level of spatial similarity in these metrics then provides information towards the phenomena driving those dynamics. Initially, to demonstrate the potential in spatially analysing RQA (or other) time series metrics, we choose a fine spatial resolution with which to carry out this analysis. By selecting one in four node points from the discretisation used for numerical simulation, this results in a spacing of 0.4 mm between the locations for which a time series is considered available. We later discuss the effect of coarser spatial resolutions.

To quantify spatial similarity, we use the spatial autocorrelation measure known as Moran’s I^44^. Given a weight matrix, *W*, that defines how different spatial locations relate to one another, this measure is defined

$$\begin{aligned} I = \frac{N}{\sum _{i} \sum _{j} w_{ij}} \frac{\sum _{i} \sum _{j} w_{i j}\left( x_{i}-\bar{x}\right) \left( x_{j}-\bar{x}\right) }{\sum _{i}\left( x_{i}-\bar{x}\right) ^{2}}. \end{aligned}$$

Here *N* is the total number of spatial sites, and *x* is the quantity for which the spatial correlation is being calculated. $\bar{x} = \frac{1}{N} \sum _i x_i$ is the mean value of this quantity, and $w_{ij}$ are the elements of the matrix *W* each describing the level of relationship between sites *i* and *j*. So long as the weight matrix is chosen such that sites close together are weighted strongly and distant sites are weighted weakly, a Moran’s I value close to the maximal value of $+1$ corresponds to large regions of similar values of the quantity of interest, while a value close to the minimum value of $-1$ corresponds to sharp spatial variation in the quantity (negative correlation). A value of zero represents independence between sites. In this work, the weighting matrix is defined by the Moore neighbourhood (surrounding eight points including diagonals), with these neighbours being given full weight and other points given zero weight,

$$\begin{aligned} w_{ij} = {\left\{ \begin{array}{ll} 1 &{} j \in \text{ Moore } \text{ neighbourhood } \text{ of } \text{ i } \\ 0 &{} \text{ otherwise }. \end{array}\right. }. \end{aligned}$$

As each site is not its own neighbour, the diagonal elements of the weight matrix $w_{ii} = 0$. As an example, given a set of values *x* for sites arranged in a 3$\times \!$3 grid, we would have a 9$\times \!$9 matrix given by

$$\begin{aligned} \begin{matrix} x_1 \, &{} \, x_2\, &{} \, x_3 \\ &{} &{} \\ x_4 \, &{} \, x_5\, &{} \, x_6 \\ &{} &{} \\ x_7 \, &{} \, x_8 \, &{} \, x_9 \end{matrix}\,\,, \qquad \qquad \qquad W = \begin{pmatrix} 0 &{} 1 &{} 0 &{} 1 &{} 1 &{} 0 &{} 0 &{} 0 &{} 0 \\ 1 &{} 0 &{} 1 &{} 1 &{} 1 &{} 1 &{} 0 &{} 0 &{} 0 \\ 0 &{} 1 &{} 0 &{} 1 &{} 1 &{} 1 &{} 0 &{} 0 &{} 0 \\ 1 &{} 1 &{} 1 &{} 0 &{} 0 &{} 0 &{} 1 &{} 1 &{} 0 \\ 1 &{} 1 &{} 1 &{} 0 &{} 0 &{} 1 &{} 1 &{} 1 &{} 1 \\ 0 &{} 1 &{} 1 &{} 0 &{} 1 &{} 0 &{} 1 &{} 1 &{} 1 \\ 0 &{} 0 &{} 0 &{} 1 &{} 1 &{} 1 &{} 0 &{} 1 &{} 0 \\ 0 &{} 0 &{} 0 &{} 1 &{} 1 &{} 1 &{} 1 &{} 0 &{} 1 \\ 0 &{} 0 &{} 0 &{} 0 &{} 1 &{} 1 &{} 0 &{} 1 &{} 0 \end{pmatrix}. \end{aligned}$$

## Results

### RQA measures encode key facets of localised activation patterns

**Figure 1:**
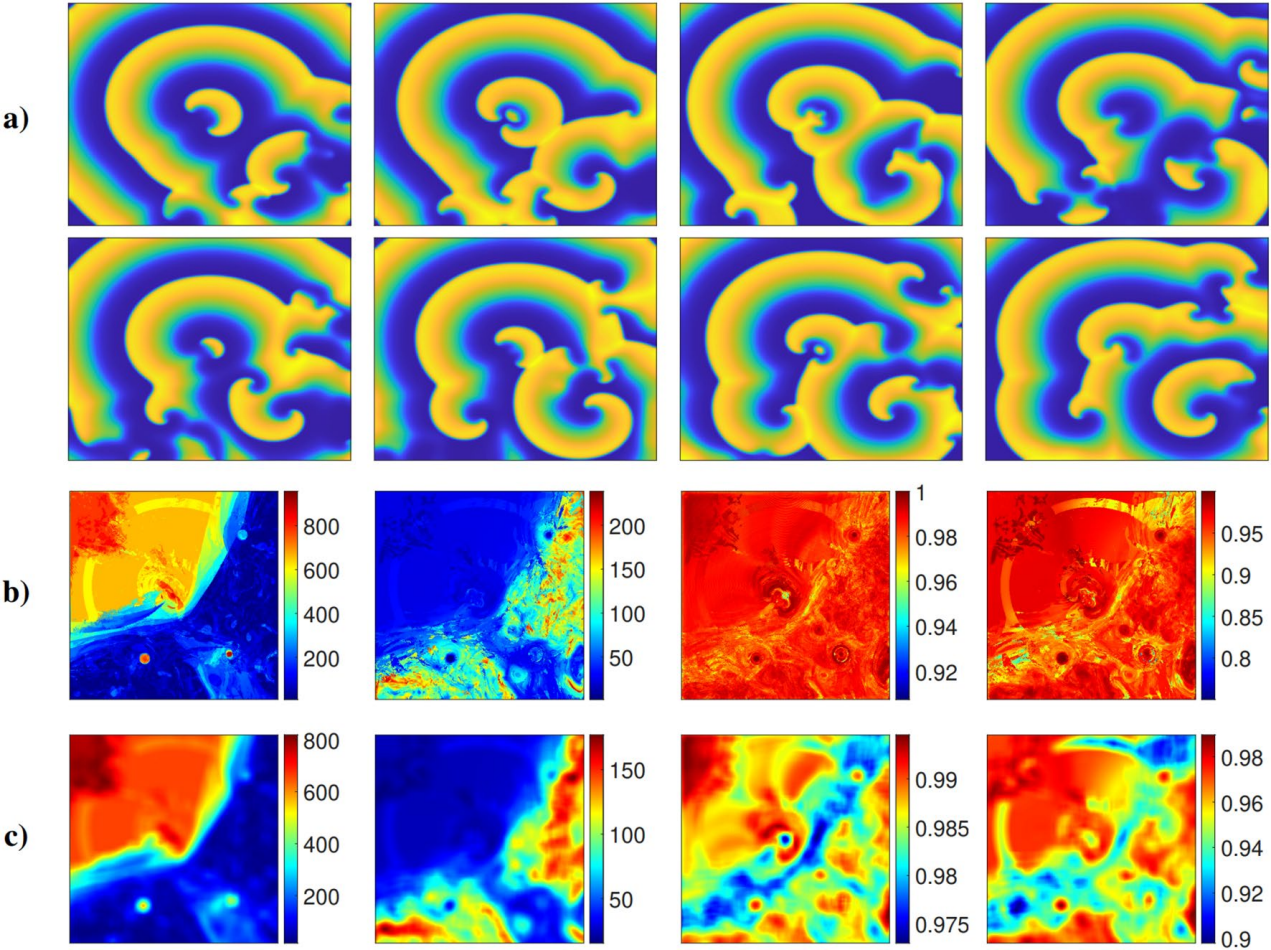
(**a**) Membrane potential snapshots, spaced by 143 ms. Regular fast pacing in the centre of a large ($12\!\times {12}\,{\text{cm}}$) slice of tissue creates regular waves radiating out toward the upper left corner. A spiral persists in the bottom right corner, and transient rotors appear in the lower left and upper right corners. (**b**) Tissue maps for the RQA measures from left to right, $L_{max}$, *RATIO*, *DET*, and *LAM*. $L_{max}$ takes on high values where activation is strictly regular, in the upper left corner and where rotor cores persist. *RATIO* highlights similar regions (via lower values), but identifies both rotor tips and the regular activation dynamics surrounding them. Measures *DET* and *LAM* highlight (via lower values) the regions where activation dynamics are most chaotic, characterised by wavelets that form where the main propagating waves interact. *DET* emerges as most appropriate for this purpose, as *LAM* also reaches low values around the rotor tip in the lower right corner. (**c**) Blurring the original maps via 2D convolution (see main text) makes the regions identified by the different RQA measures more visually distinguishable. In particular, the boundaries between regions of regular and fibrillatory activation detected by measure *DET* become much more pronounced.

We first analyse how the range of RQA measures we consider respond to different patterns of activation. For this purpose, we simulate the complex response of cardiac tissue to very fast pacing (further details in Methods), generating regions that experience regular activation, regions that experience irregular activation, and yet more intricate dynamics where these two phenomena interact (Fig. 1a).

Regular waves propagate outwards from the centre of the tissue (the location of the pacing stimulus), and continue undisturbed out towards the upper left corner. In the lower right corner, a stable rotor develops and persists throughout the simulation period. Two more transient rotors appear during the course of the simulation, in the lower left and upper right corners. The remainder of the tissue experiences the interactions of the different waves or wavelets created by these different rotors and the pacing site, exhibiting irregular patterns of activation and AP propagation characteristic of fibrillation.

We analyze the RQA measures by calculating their values for the time series of the (dimensionless) membrane potential, *u*, at many sites throughout the tissue, producing spatial maps of these measures. These maps, some of which are displayed in Fig. 1b, are then compared with the spatially varying activation dynamics through the tissue. We also consider smoothed versions of these spatial maps, obtained by averaging the value of each pixel and its immediate neighbours (the 2D convolution principle). This reduces noise and better highlights differences in value between larger-scale regions of the tissue (Fig. 1c).

The RQA measure $L_{max}$ best detects the regular waves propagating from the centre to the upper left corner, and the rotor tips at the bottom of the simulation domain. The measure *RATIO* acts somewhat oppositely to $L_{max}$, taking on higher values where the activation pattern is disordered. In contrast with $L_{max}$, however, regions where activation is quite regular but does not occur at a single, fixed frequency are still labelled as ordered (such as the lower right corner of the domain). As such, *RATIO* is less sensitive to regularity violations than $L_{max}$.

The measures *DET* and *LAM* are the least sensitive to regularity violations. *DET* takes on high values (greater than 0.9) throughout the entirety of the tissue, but after spatial smoothing, it becomes visually clear that this measure takes lower values where the dynamics are the least predictable. That is, the portions of the tissue where the activation waves from the different rotors collide and interact with each other. *LAM* performs a similar role, largely matching *DET* in terms of where it takes on a relatively low or high value.

These different sensitivities help to guide appropriate selection of RQA measure(s), depending on the investigation being conducted. For example, *DET* and *LAM* are best suited for identifying downstream regions of wavebreak, unlikely to house any persistent drivers of arrhythmia. On the other hand, in the following section, we demonstrate the use of the most sensitive measure $L_{max}$, along with a second RQA measure, to very accurately identify locations of stable functional re-entry.

### Detection of rotor tips

As Fig. 1 demonstrates, persistent functional re-entry can manifest in more chaotic, fibrillatory dynamics away from the rotor location. Such persistent re-entries are often termed “mother rotors”, and are one of the prominent theories of arrhythmias not driven by some external circumstance (for example structural heterogeneity due to scarring)^45^. Others argue that all rotors are liable to appear and disappear, and that the dynamics behind these arrhythmias are better interpreted as a stochastic process of creation and annihilation^46^. Regardless, should such stable, self-sustained spiral waves exist, their accurate detection is critical for informing interventions such as ablative surgery. Automatic detection of rotors using spatial information remains a topic of interest, although has concentrated more on identification of any spiral wave tips (including both sustained rotors and transient tips appearing in fibrillation)^47^. Here we consider the use of RQA metrics specifically for the detection of sustained, spatially-fixed rotors using only local time series information.

As discussed previously, $L_{max}$ serves as a very sensitive measure of regularity in a site’s membrane potential time series, and is thus an important component in the detection of spiral waves. However, to assess the fixation of these spiral waves, we also involve the RQA measure $L_{mean}$. By indicating the average length of diagonal lines in the RP, $L_{mean}$ is related to the mean prediction time^48^ and helps to distinguish between persistent spiral waves and other regular dynamics, such as persistent stimulation due to waves of activation emanating from a site of regular pacing.

**Figure 2:**
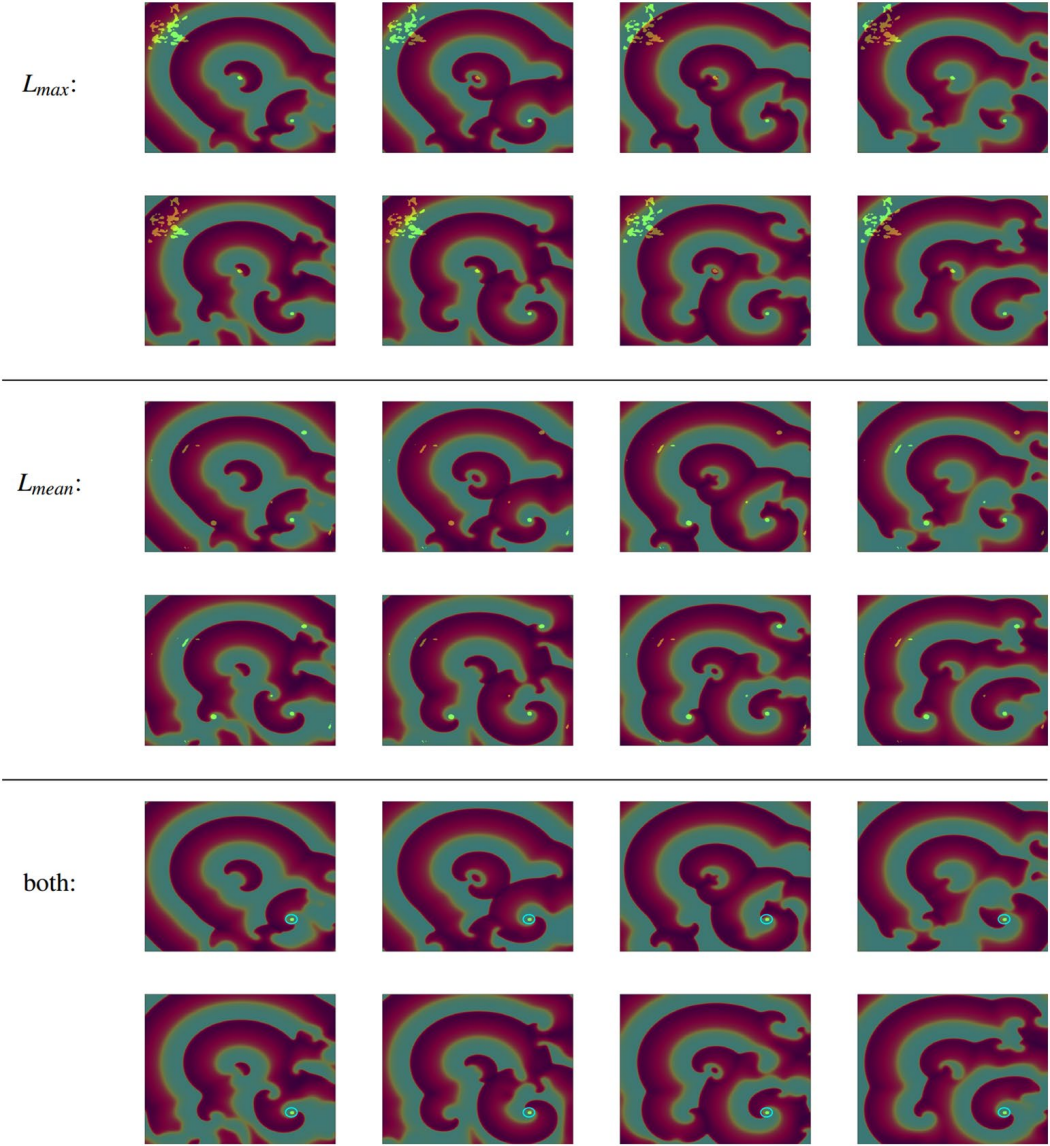
Snapshots (spaced by 143 ms) of AP propagation, with regions for which the membrane potential timeseries produces an RQA measure ($L_{max}$, $L_{mean}$, or both at the same time) exceeding a threshold value highlighted in bright green. These measures correspond to the duration of patterns of periodic activation (indicated in an RP by the length of diagonal lines), and as such the value of the $L_{max}$ threshold selects how long a regular activation pattern must persist to be identified. In this case, the threshold value $L_{max} \ge 800$ detects only the tip of the spatially pinned rotor, when combined also with a threshold $L_{mean} > 15$ (fifth and sixth rows, highlighted region in the lower right corner within the light blue circle).

To enable automatic detection using these measures, we select restrictive thresholds for their values that pick out only the most evident examples of the types of dynamics they characterise. The regions identified using threshold values for these two RQA measures are shown in Fig. 2, together with the intersection of these two sets of regions that serves as a detector of stable rotor cores. Here, this identifies the rotor in the bottom right, the only one that persists throughout the pictured time window in the simulation. The transient rotors that form, but are then annihilated, fail to reach the critical $L_{max}$ value. Meanwhile, regions of highly regular dynamics not reflective of a rotor are removed by failing to meet the threshold value of $L_{mean}$.

Notably, as the value of $L_{max}$ represents the longest period of time the system remains in a periodic pattern, the threshold value selected for this RQA measure also reflects how long a rotor must persist before it will be labelled as stable by the detection process. This provides a degree of control over the detection process to the user. As we demonstrate in Figure S1, reducing the threshold value of $L_{max}$ presents a means of also identifying regions that are occupied by a stable rotor for a reduced amount of time.

Considering the spatial maps seen in Fig. 2, a further potential role for RQA in the detection of sustained rotors emerges when there is even limited access to spatially-distributed time series data. As discussed in the previous section, RATIO serves as a measure of irregularity, that is less sensitive to minor disruptions of a periodic activation pattern. This allows it to identify (with low values, indicating regularity), the region immediately surrounding the stable rotor (second column in Fig. 2b,c). Distant from the rotor (top left), the RATIO value is similarly low, as this region is also activated in a regular fashion. The key feature distinguishing a stable rotor, at least in the presence of fibrillation, is a region of low RATIO surrounded by considerably higher values, and this could be detected by RQA applied to only a few time series collected from locations with the appropriate spatial separation.

### Use of RPs of AP traces to classify arrhythmia dynamics

**Figure 3:**
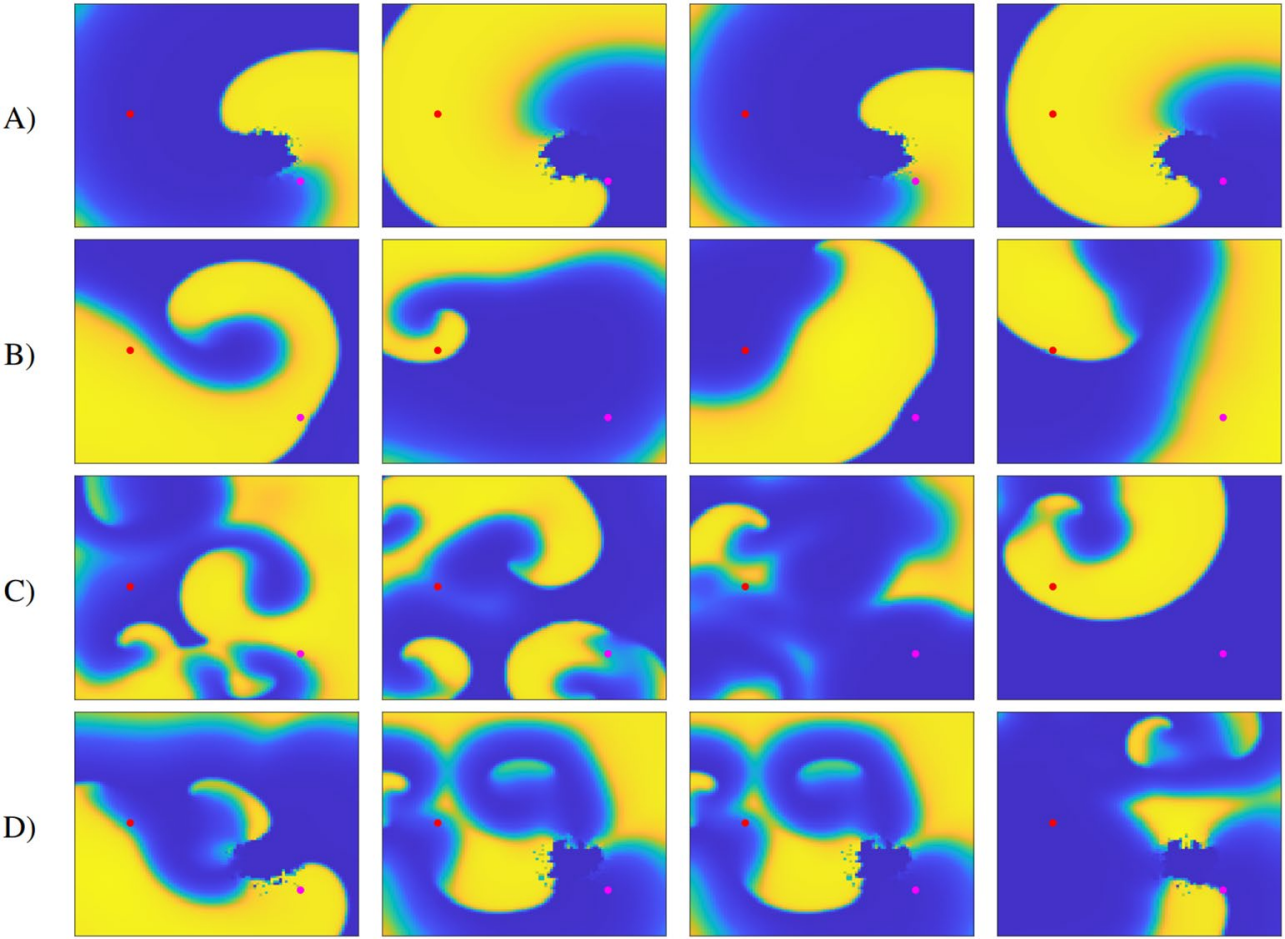
Snapshots (spaced by 100 ms) of each group of AP propagation modelled on a smaller tissue square (with the size of $4\times {4}\,{\text{cm}}$). At each tissue site, the time series is analyzed using RQA, OI, RP eigenvalues and entropy. Examples of individual time series at the points in red and magenta are given in Fig. 4. The dynamic parameters of these time series are then used to classify individual propagations. (**A**) A spiral wave anchored to a highly fibrotic region resulting in sustained re-entry. (**B**) Meandering spiral wave. (**C**) Tissue experiencing fibrillatory activation. (**D**) Spiral wave anchored to a scar region (bottom right), that breaks up into wavelets away from the main rotor.

We next consider a series of simulations using smaller slices of tissue (comparable to the size of the human heart atrium), each including a region of scarring of varying size and severity. In contrast to the study described in previous sections, characterised by a range of different dynamics and their interactions, these simulations using a smaller domain produce activation dynamics corresponding to tachycardia or fibrillation that can be classified on the tissue scale (Fig. 3).

**Figure 4:**
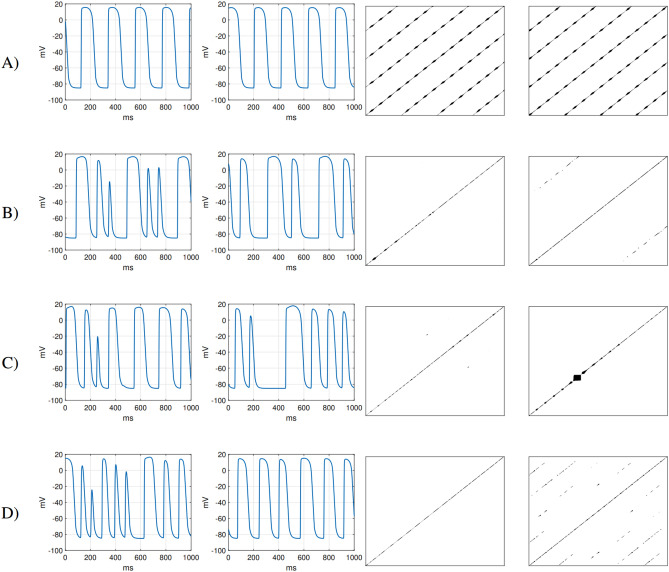
Membrane potential traces from two distinct locations (depicted in Fig.3) in the tissue (left side) and its recurrence plots (right side). (**A**) A spiral wave anchored to a highly fibrotic region resulting in sustained re-entry. The spiral wave regularly stimulates the tissue at both sites. This phenomenon is manifested in RPs by long uninterupted diagonal lines. On these diagonals there are small squares created by the plateau phase. (**B**) The regular meandering of the spiral wave manifests by repeating patterns in the time series. Due to the long period, uninterrupted lines are not visible in the RP as in the case of an anchored spiral wave. (**C**) Tissue experiencing fibrillatory activation. The time series created by this activation does not contain any regular patterns. Therefore long uninterrupted lines are not visible in the RP. The large black square visible in the right RP is due to the membrane potential remaining in the resting state between 200 and 400 ms (in the right time series). (**D**) Spiral wave anchored to a scar region (bottom right), that breaks up into wavelets away from the main rotor. This propagation manifests irregular patterns (left time series) and regular AP waveforms (right time series). However, the surrounding fibrillatory activation disrupts the regularity of these waveforms. As a result, the diagonal lines are interrupted.

For each simulation, RPs are generated from the membrane potential time series at different locations using the approach described in the Methods. The time lag and embedded dimension are found using the *nonlinearTseries* package for the R programming language^49^. These parameters are used for phase space reconstruction. According to Takens theorem^50^, it is possible to use the time series that comes from a dynamic system to reconstruct a trajectory using time lag and embedding. Reconstructed trajectories will have the same dynamic properties as the original dynamic system. An RP is then constructed from these trajectories. For example, the time series $X_1, X_2, X_3, \dots , X_{10}$ with time delay 3 and embedding dimension 2 will be reconstructed into a two-dimensional phase space as $(X_1,X_4),(X_2,X_5),( X_3,X_6),\dots ,(X_7,X_{10})$. The *nonlinearTseries* package computes the time lag using the first zero crossing of the autocorrelation function, and the embedding dimension using L. Cao’s algorithm^51^. The threshold $\epsilon$ for recurrence detection is determined to be 3% of the range of the analyzed time series. The resultant RPs are pictured in Fig. 4, where they are seen to capture successfully some of the features of the different types of activation pattern.

#### Tachycardia driven by a stable rotor

Where the re-entry anchors to the region of fibrosis, and the wave does not exhibit breakup away from the rotor core, this produces a regular but tachycardic pattern of activation, see Fig. 4a. AP morphology is consistent throughout the tissue (except the damaged site), similar to sinus rhythm, except for the pacing frequency. The RPs in this case consist of diagonal lines that reveal the periodic nature of activation. Patterns are superimposed on these lines at regular intervals, created by the portions of the time series where the tissue is at rest.

#### Tachycardia driven by a wandering rotor

Re-entries that do not fix to a heterogeneity in the tissue and do not produce wavebreak also result in tachycardic activation of the tissue. The path of the rotor tip follows a twisting pattern, resulting in inconsistent lengths of time between activation events and as a result, differences in morphology between individual APs, see Fig. 4b. RPs associated with these dynamics do not show the many regularly spaced diagonal lines indicating periodicity. However, as each completion of the rotor’s twisting path does represent a single period, diagonal lines may still appear far away from the main diagonal, with consistent spacing.

#### Fibrillation driven by wavelets

Fibrillation may also be sustained by many individual wavelets, created and annihilated by their unpredictable interactions with each other, see Fig. 4c. In these circumstances, membrane potential time series show highly varied AP morphology and activation timing, resulting in RPs consisting only of lines along the main diagonal, possibly with distinct blocks corresponding to prolonged periods of rest (example in Fig. 4).

#### Fibrillation driven by a stable rotor

Wave break can occur distant from an anchored spiral wave re-entry, resulting in disordered activation of the surrounded tissue, see Fig. 4d. This results in very distinct membrane potential time series depending on the region of the tissue from which readings are taken. RPs distant from the anchored re-entry are chaotic, consisting mostly of a line along the main diagonal. In contrast, RPs at the location of the spiral wave show many evenly-spaced diagonal lines, characteristic of consistent, regular activation. Owing to minor disruptions by the surrounding chaotic dynamics, however, these lines are interrupted in a fashion not seen for tachycardia driven by a stable rotor.

Using the membrane potential time series from just two locations, each different class of arrhythmic dynamics can be identified using the RPs leveraging the summarised distinguishing features. However, this relies upon qualitative observation and an appropriate selection of the locations to ensure capture of both regularly activated and irregularly activated tissue, in the case both are present. Such important locations will not generally be known prior to data collection. Consequently, we now examine whether these different arrhythmia dynamics on the tissue scale can be identified automatically, using the membrane potential time series recorded at many points throughout the tissue.

### Spatial behaviour of metrics for different arrhythmia mechanisms

To achieve our goal of distinguishing these four important types of arrhythmia in an automated fashion, we first consider how a large range of RQA metrics, along with OI and entropy as other measures of time series regularity, vary spatially throughout the tissue. We rely on the sophistication of these metrics to capture the intricacies of the different activation patterns and use relatively simple means to characterise this spatial variation. Specifically, we consider the mean value of each metric across all measurement locations in the tissue and the spatial correlation of each metric as evaluated by Moran’s I. This provides an interpretable and lower-dimensional model for the automatic classification we will suggest.

**Figure 5:**
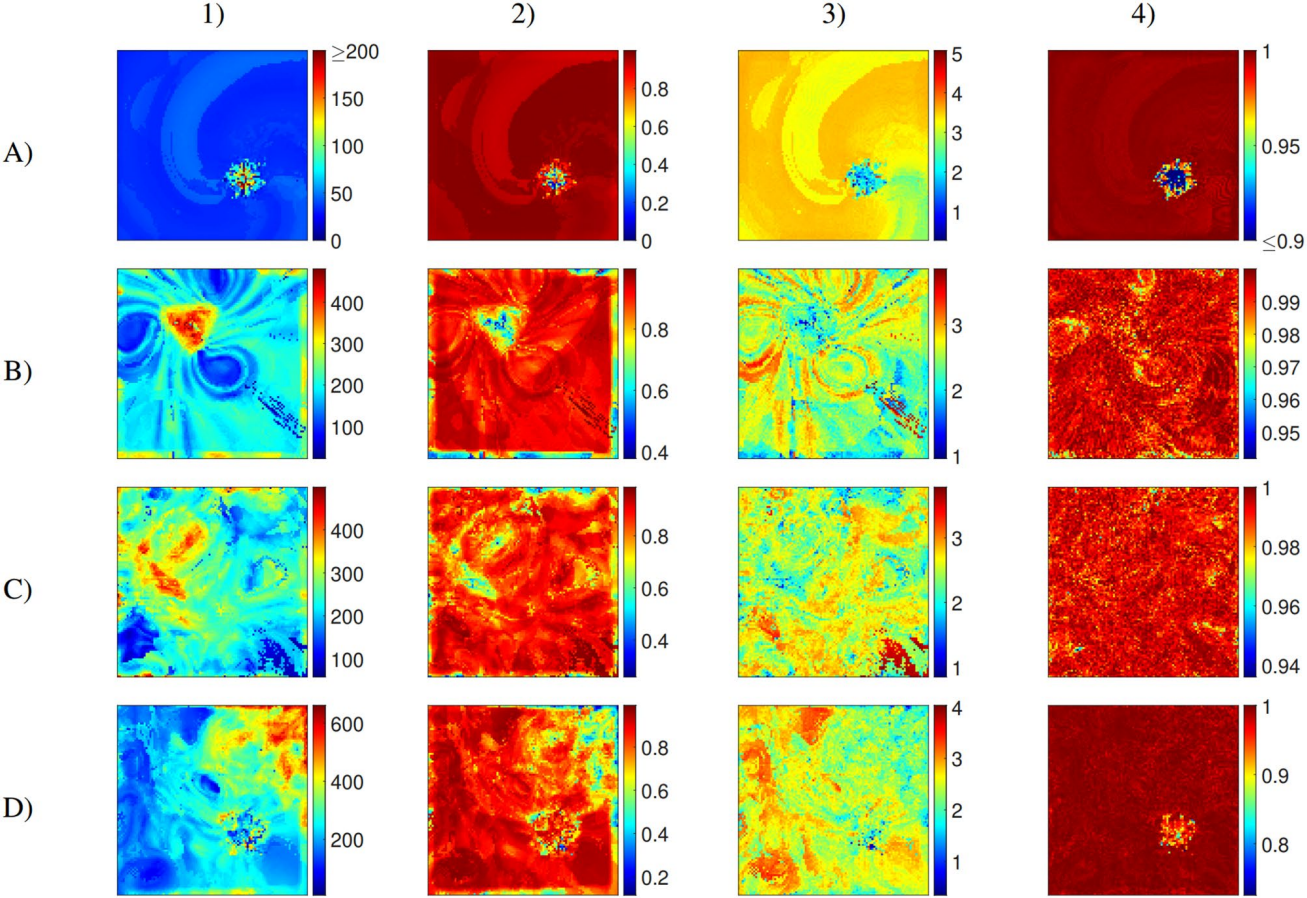
Example of calculated tissue maps (with the size of $4\times {4}\,{\text{cm}}$) depicting the spatial distribution of selected RQA measures. Rows: **A**) anchored rotation, **B**) traveling rotor, **C**) fibrillation, **D**) fibrillation with anchored rotation. Columns: RQA measures 1) *RATIO*, 2) *LAM*, 3) *ENTR*, and 4) *DET*. A pattern indicating the type of analyzed propagation can be found in all maps. In anchored rotation (A), a spiral created by the rotational movement of the AP can be seen. Three spirals formed by the meandering rotor can be found (**B**) (in each spiral, the AP re-entries the tissue). The region around which the AP rotates is highlighted in the middle of these spirals. Fibrilatory activation (**C**) is manifested in maps by irregular, chaotically distributed shapes. Fibrillation associated with anchored rotation (**D**) in the maps (especially in measure *RATIO*) shows larger contiguous regions created by regular propagation made by anchored rotation. If we compare the mean values of individual RQA measures, we can see a lower mean value of *RATIO* (Ratio between *DET* and *REC*) for anchored rotation (see boxplots in Fig. 6). This phenomenon is caused by the higher value of the *RR* measure due to diagonal lines manifesting periodic motion in RP (see Fig. 4). There is also a high mean value for measure *LAM* (percentage of vertical lines in RP). A regular resting phase causes this phenomenon during anchored rotation (manifesting as black squares in RP). This phase can also be partially found in the meandering rotor, as its average value in the tissue is the second highest (see Fig. 6). The average value of the *ENTR* measure is the highest for anchored rotation. *ENTR* achieves high values, as RPs of this type of propagation are the most complex (due to diagonal lines and their connected patterns). The Mean value of the *DET* measure is highest for anchored rotation due to the repetitive nature of this propagation. The irregularity of fibrillatory activation is reflected in the lowest value of this measure for all the types of analyzed propagation.

In Fig. 5, spatial maps of some of the different RQA measures are displayed, demonstrating how each can be used to identify or distinguish the features of the different types of arrhythmia. Across all the metrics, tachycardia driven by localised re-entry results in values of the metrics that are spatially homogeneous, suggesting that very high values of spatial correlation may identify this pattern of behaviour. In the case of a rotor in a wandering orbit around a location, this region is highlighted by both RATIO and LAM. Some metrics also produce multiple teardrop shapes around this region, indicating the points where the rotor tip turns around to continue its orbit.

In fibrillatory arrhythmias, the spatiotemporal chaos results in far more irregular spatial maps of the different RQA metrics. When fibrillation is driven by an anchored rotor, larger contiguous regions of similar values appear, suggesting the possibility that spatial correlation might allow the two different causes of fibrillation considered here to be distinguished. To explore this, we now consider how our summarisation measures for the spatial RQA maps vary within a larger set of simulations, thus demonstrating the potential for automated classification. We give a detailed characterisation of a number of our measures in Table 1.

### Classification of arrhythmic patterns in cardiac tissue

**Figure 6:**
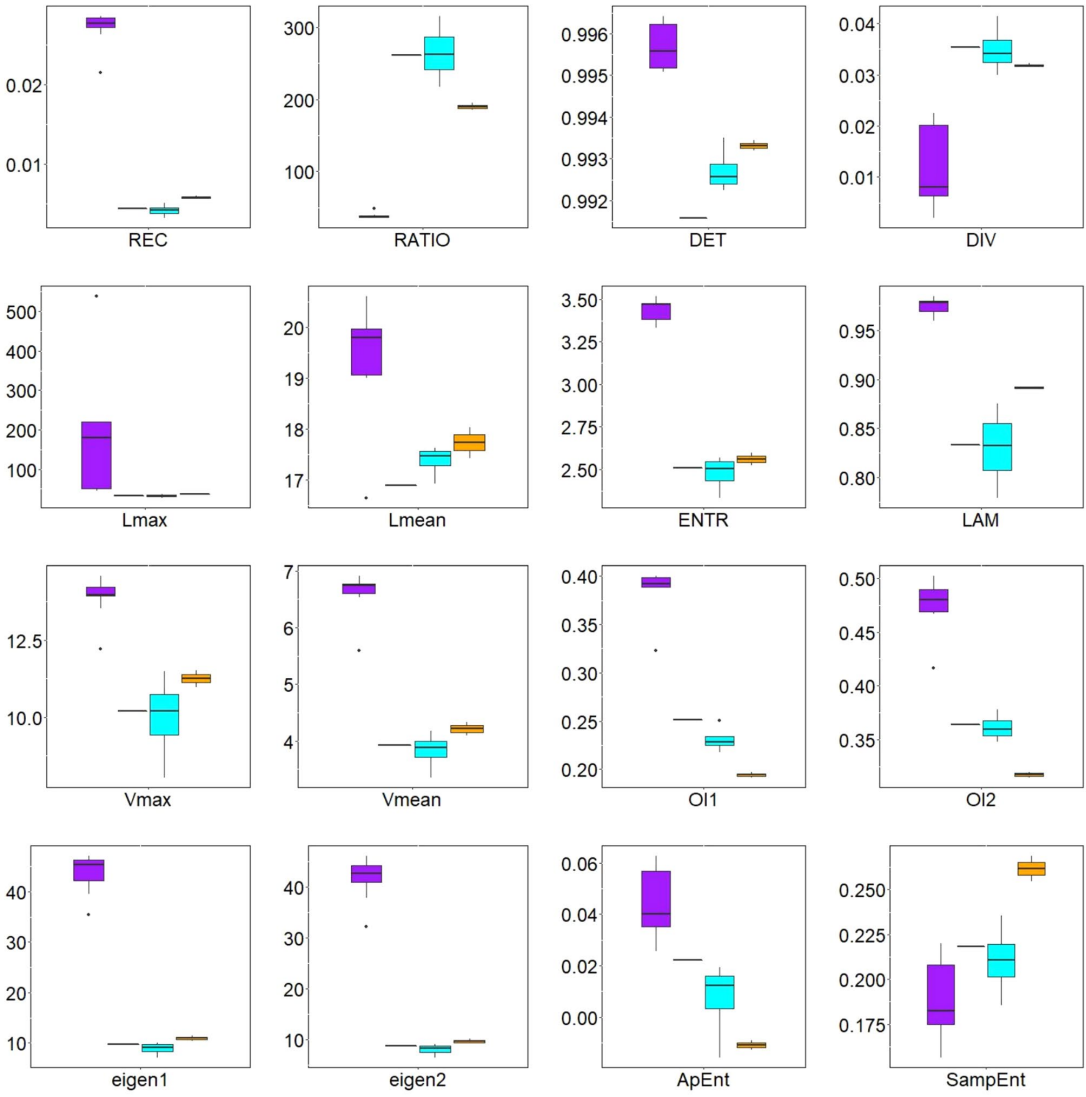
Boxplots of mean RQA measures for anchored rotation (1st box, purple), fibrillation with anchored rotation (2nd box), fibrillation (3rd box, blue), and travelling rotor (4th box, orange). Depicted values are the mean value of the RQA measure, averaged across spatial locations throughout the tissue. The most distinguishable of these is anchored rotation, with almost all measures distinguishing this type of propagation. The travelling rotor is also easily differentiable. This propagation can be distinguished from other types by *RATIO*, *LAM*, *SampEnt* and both OIs. It is most challenging to differentiate between fibrillation and fibrillation with anchored rotation. However, these can be distinguished by *DET* and $L_{mean}$. Detailed description of all depicted boxplots is given in Table 1.

**Figure 7:**
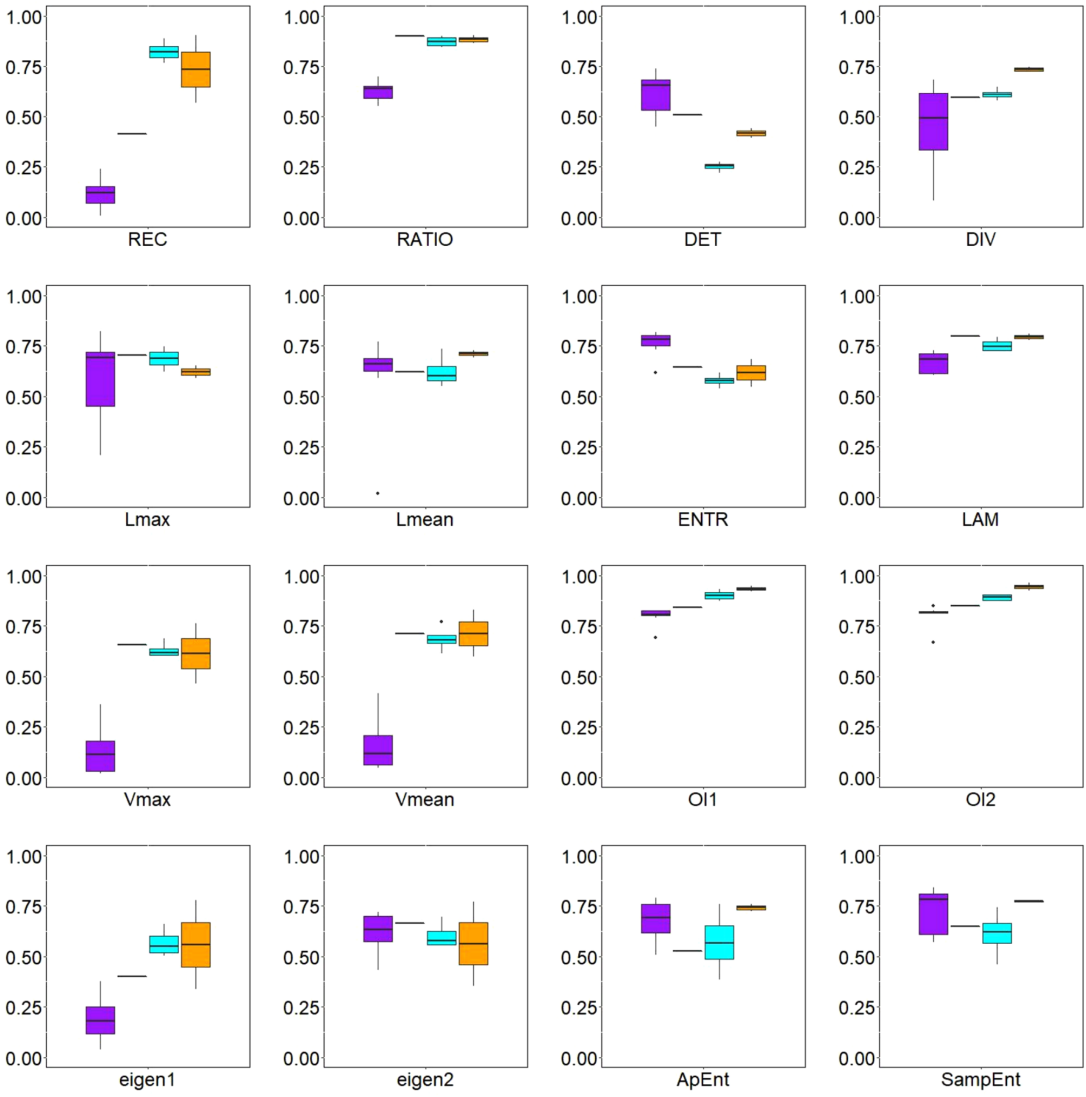
Boxplots of spatial correlation between values of different RQA measures throughout the tissue, as measured by Moran’s I, for anchored rotation (1st box, purple), fibrillation with anchored rotation (2nd box), fibrillation (3rd box, blue), and travelling rotor (4th box, orange). Anchored rotation can best be distinguished using Moran’s I. The most valuable benefit of this analysis can be seen in measures *REC* and *DET*. These measures indicate fibrillatory activation (*DET*) and fibrillation with anchored rotation (*REC*). In most other cases, Moran’s I reach similar values.

In Figs. 6 and 7, boxplots indicate how the spatial summaries of a wide range of metrics vary across simulations of the different classes of arrhythmia. Numerical values for the four cases used as examples (those depicted in Figs. 3, 4 and  5) are also given in Supplementary Tables S2 and S3. Examining these results, some of the RQA-derived metrics emerge as compelling choices for distinguishing fibrillation from tachycardia, and identifying the presence of anchored re-entry. From these figures, we see that it is straightforward to distinguish between anchored rotation and fibrillation, which any of the presented metrics can differentiate. In particular, the repetitive nature of tachycardia driven by an anchored rotor results in much higher mean values for REC. Mean values for RATIO and LAM also capture this distinction whilst differentiating the next most regular type of arrhythmia (tachycardia driven by a wandering rotor) from the fibrillatory cases.

Across the many metrics considered, none except perhaps DET can clearly identify the driver for fibrillation using its mean value. This is the motivation for also incorporating a measure of spatial correlation, where the spatial correlation of both REC and DET emerge as the most compelling means of separating fibrillation driven by a fixed “mother” rotor or by many transient wavelets. The most complicated aspect is distinguishing combinations of fibrillation and anchored rotation (this last pattern most often reaches the same statistical metric values as pure fibrillation). This pattern can be indicated by the mean value of the RQA measure *DET* and the spatial correlation of *REC* (for this metric a gradual decrease can be observed as fibrillation passes into anchored rotation). The travelling rotor also achieves similar values in the measures as in fibrillation. However, it can be distinguished from this type of pattern mainly by the spatial correlation of the RQA measure of *DET*.

**Figure 8:**
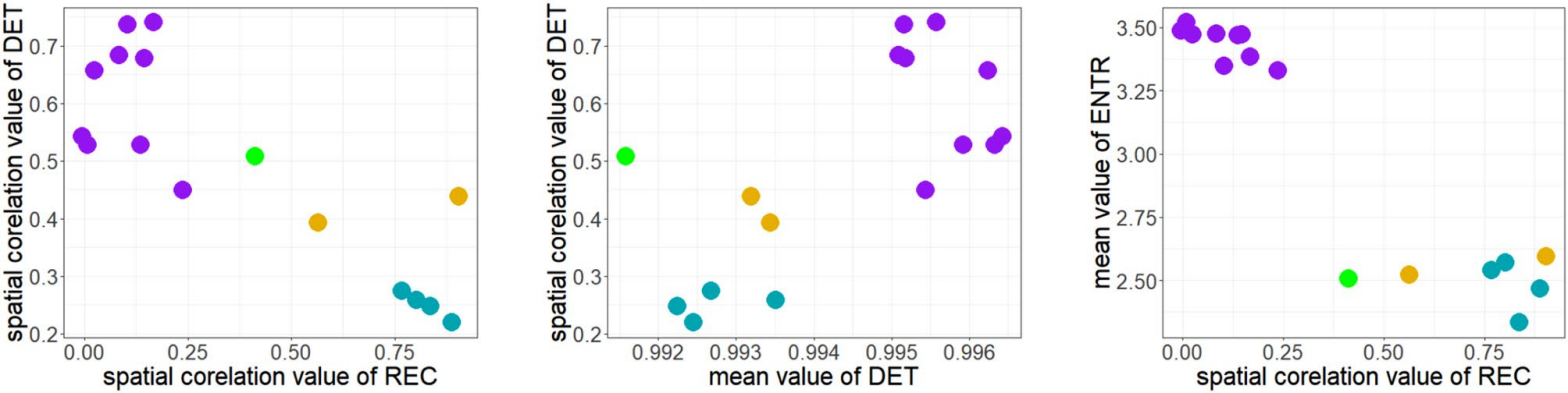
Scatter plots of selected parameters (fibrillation-blue; anchored rotation-purple; fibrillation with anchored rotation-green; traveling rotor-orange). These plots show the classification of individual types of propagation into clusters. Although Moran’s I achieved similar values for most RQA measures, the evaluation of *REC* and *DET* using this method contributed significantly to this clustering.

In Fig. 8, we demonstrate how by taking the spatial correlation of one of these RQA measures, together with an appropriately chosen second summary measure of the spatial RQA map, clear clustering of the different types of tachycardia and fibrillation is achieved. Anchored rotation can be easily distinguished from other types of cardiac arrhythmia. As can be seen in Fig. 6, this fast and regular motion of AP is differentiable using the mean value of almost any RQA measure in the tissue and spatial correlation of RQA measures *REC* and *DET* (see Fig. 7). Separation of other activation patterns is less straightforward but can be achieved, primarily through Moran’s I of *REC* and *DET* (Fig. 8).

DET is the overall most promising quantity for this purpose of distinguishing different types of arrhythmia. In essence, it serves as a measure of the predictability of a system. In this case, although the monodomain model provides a deterministic description of how electrical activity in the tissue evolves, each “system” being analysed via RQA is a single spatial point in the tissue. The dynamics at individual points consist of deterministic behaviour defined by the ionic model together with a component that is non-deterministic in terms of only local information, namely the diffusive transfer of electric potential in/out of the site. The dominance of the deterministic component manifests in values of DET close to unity in the bulk of the tissue, and the mean value is highest for anchored rotors as their regularity makes the diffusive transfer of membrane potential also become essentially deterministic. Where a rotor persists, even if it meanders, this creates distinct regions of higher or lower DET, which are then detected by the Moran’s I value for this metric. In the self-sustaining chaos of fibrillation not driven by a rotor, although all points in the tissue become functionally the same, the variance-normalized nature of Moran’s I allows it to correctly detect chaotic fluctuations around some baseline value. Self-sustaining fibrillation is thereby characterised by particularly low values of spatial correlation of DET.

The first two scatterplots in Fig. 8 demonstrate that it is possible, using only two different measures, to differentiate between the different activation patterns. Adding another measure should make it possible to separate these clusters even further and thus would be expected to improve performance of a classifier. Given the strong visual separation of each class of dynamics, automatic classification with widely-used techniques such as *k*-means^52^ or support vector machines^53^ may be feasible. Of course, a full demonstration of classification accuracy would require a more numerous set of simulations with good representation of each class, something difficult to achieve here due to the high rarity with which some behaviours (in particular fibrillation driven by a stable rotor) were observed.

**Table 1:** Detailed description of boxplots showing the distribution of analyzed measures in Figs. 6 and 7.

$\textrm{REC}$: Regular anchored rotation is reflected in the high average value of *REC*. The value is also slightly increased for the traveling rotor (see Fig. 6 and Supplementary Table S2). The spatial correlation of this measure is low for anchored rotation (as opposed to fibrillation and travelling rotor). Spatial correlation can also distinguish the combination of fibrillation and anchored rotation from other types of propagation (see Fig. 7 and Supplementary Table S3)	
$\textrm{RATIO}$: This measure reaches a low value for anchored rotation. The measure is also able to distinguish a traveling rotor from fibrillation, including its combination with anchored rotation (see Fig. 6 and Supplementary Table S2). The spatial correlation reaches lower values for anchored rotation (see Fig. 7 and Supplementary Table S3). In the traveling rotor, *RATIO* reaches high values in regions between rotor meandering (see Fig. 5)	
$\textrm{DET}$: In Fig. 6 and Supplementary Table S2 it can be seen that the regular rotational movement of the AP around the scar tissue reaches high values of *DET*. On the contrary, chaotic fibrillation results in a reduction in its value (but these differences are very small). This situation is also reflected in the spatial correlation of this measure (see Fig. 7 and Supplementary Table S3). This phenomenon can be also noticed in Fig. 5. In this figure an even distribution of *DET* in the map for AP anchored rotation propagation is shown as opposed to other propagation types	
$\textrm{DIV}$: Anchored rotation reaches a reduced average value. The spatial correlation of this measure cannot distinguish between different types of AP propagation	
$\mathrm {L_{max}}$, $\mathrm {L_{mean}}$, and $\textrm{ENTR}$: regular movement of the AP around the scar tissue results in a slightly increased value of these measures for anchored rotation. The spatial correlation of this measure cannot distinguish between different types of AP propagation	
$\textrm{LAM}$: the average value of *LAM* is increased not only for anchored rotation but also for the travelling rotor. As a result, these two types of APs can be distinguished from propagation where fibrillation is present. Spatial correlation is comparable for all types of APs. RQA maps of individual AP propagations are very similar to the measure *RATIO*, the region around which the traveling rotor meanders is highlighted (see Fig. 5)	
$\mathrm {V_{max}}$, and $\mathrm {V_{mean}}$: anchored rotation can be very well distinguished by both the mean value (see Fig. 6 and Supplementary Table S2) and the spatial correlation (see Fig. 7 and Supplementary Table S3). Other types of AP propagation cannot be distinguished from each other	
$\textrm{OI1}$, and $\textrm{OI2}$: using the mean value, the traveling rotor and anchored rotation can be well distinguished (see Fig. 6 and Supplementary Table S2). Regular rotation results in a large amount of power placed in the first two highest peaks in the frequency spectrum. This phenomenon is manifested in high *OI*1 and *OI*2 anchored rotation. In contrast, the very low value of these measures for the traveling rotor indicates irregular AP propagation. Due to the analysis of the short time series of the modeled AP (1 s), this irregularity is not observed. Spatial correlation of these measures indicates the ability to distinguish between all analyzed types of AP propagation (see Fig. 7 and Supplementary Table S3). These differences are relatively small	
$\textrm{eigen1}$, and $\textrm{eigen2}$: the mean value of the first and second highest eigenvalue of the recurrence plot for anchored rotation ($<40$) achieves the highest discrimination from other AP types ($>10$) among all measures tested. Other types of AP propagation can also be distinguished using this measure. However, the differences in the mean values of eigen1 and eigen2 between these types of propagation are minor (approximately 1). Spatial correlation of these measures provides distinction only for anchored rotation measured at *eigen*1	
$\textrm{ApEnt}$, and $\textrm{SampEnt}$: the mean approximate entropy calculation is able to distinguish all analyzed types of AP propagation, except the traveling rotor. However, this type of AP propagation can be distinguished by *SampEnt*. *SampEnt* and *ApEnt* show the opposite direction of entropy increase. *SampEnt* reaches the lowest level for anchored rotation and the highest for travelling rotor, while for *ApEnt*, this trend is reversed (see boxplots in Fig. 6). These boxplots suggest that *SampEnt* is more appropriate for this data type. It can be assumed that regular movement of the AP around the scar should achieve less complexity than irregular fibrillation or a more complex rotor meandering. This assumption is also supported by the work of Montesinos et al.^54^ In this paper, the authors examined the time series of centres of the pressure during a posturography test of different groups of adults. They found that *SampEnt* is more appropriate to distinguish these groups. Spatial correlation of any entropy is the same for all types of APs	

### Effects of spatial resolution

The use of RQA metrics obtained from spatially-distributed time series to distinguish different classes of arrhythmia invites the question of what spatial sampling resolution is required to achieve this. The spatial mean features can be considered as estimates of the integral expectation over the spatial domain, such that their calculation at different spatial resolutions corresponds to different quadrature estimates of the same underlying quantity. In contrast, the use of an adjacency-based weight matrix in Moran’s I calculations means that a weight of unity is assigned to sites a different distance away when the spatial resolution changes. As such, the spatial correlation features change more fundamentally when the sites for which time series are available become more distant.

In practise, we find that regardless of the type of feature (mean or spatial correlation), the most useful metrics displayed in Fig.  8 begin to degrade in their ability to separate the different categories of arrhythmia as the sampling sites become more distant, as seen in Figure S2. The mean and spatial correlation of DET values still provide a pair of metrics that achieves separation for a separation of spatial sites of $\Delta _x = {1.2}\,{\text{mm}}$. Beyond this, it becomes difficult in particular to distinguish meandering rotors from self-sustaining fibrillation. The mean value of ENTR throughout the tissue remains a very good means of identifying the regular activation patterns produced by an anchored spiral wave, at least up to $\Delta _x = {4}\,{\text{mm}}$, although we expect this to be the easiest case to distinguish. Assuming that tachycardic and fibrillatory dynamics can be separated by more easily available, larger-scale approaches (consider for example the typical electrocardiogram), perhaps most interesting is the separation of fibrillation that is self-sustained, and fibrillation arising due to wavebreak away from a stable rotor. The results in Figure S2 suggest this may still be possible for time series collected from sites separated by $\Delta _x = {4}\,{\text{mm}}$, given the consistent and considerable separation of these two classes in terms of the spatial correlation of DET.

## Discussion and conclusions

The prevalence of atrial fibrillation is 0.51% of the worldwide population^55^. Although this disease does not immediately endanger patients’ lives, it significantly reduces their quality of life. In addition to pharmacological treatment, this disease can also be treated with catheter ablation with the pulmonary vein isolation ablation strategy as its gold standard^56^. Ablation using CFAEs can also be used as a supplementary treatment. This technique aims to define regions in the tissue where pro-arrhythmic phenomena occur.

We simulate the action potential (AP) propagation in cardiac tissue of two sizes. The first scenario is a tissue of $12.5\times {12.5}\,{\text{cm}}$. In this tissue, we have produced a simultaneous propagation of regular waves, irregular wave breaks, and rotational movement of AP. We show that RQA can identify these propagations and find re-entrant regions. In particular, via a combination of RQA measures $L_{max}$ and $L_{mean}$ we detect sustained, spatially fixed rotors, which are active elements in maintaining atrial fibrillation. We demonstrate how rotors with a lower duration of fixation to one location can also be detected by reducing the the required threshold for measure $L_{max}$. Our results also suggest limited spatial information (time series taken from a few surrounding points) might be enough to detect regions occupied by a stable rotor (as opposed to precise detection of its centre), at least in the context of fibrillation. Unfortunately, as the spatial map of RATIO in Fig. 5d demonstrates, this usage does not generalise to rotors anchored to a complex fibrotic obstacle, as this disrupts measures of regularity.

In the second scenario, we simulate $4\times {4}\,{\text{cm}}$ sized tissue corresponding to the size of a human heart atrium. In these simulations, we observe four types of AP propagation: rotor anchored to fibrosis, rotor moving around the tissue, fibrillation, and anchored rotor with fibrillation taking place in its vicinity. We analyze these simulations by RQA, the organizational indices (OI), and the eigenvalues associated with the recurrence plots (RP). We investigate these features by their mean value and spatial distribution using Moran’s I and show that combining these features makes it possible to distinguish these types of AP propagation in tissue. Spatial correlation has proven to be particularly useful in terms of the RQA measure *DET* (the percentage of recurrence points that form diagonal lines). Although the mean value of *DET* is almost the same for all analyzed types of propagation, their spatial correlation differs. Moran’s I reaches the highest value for anchored rotation. If fibrillation also occurs in the tissue, this value is reduced. The lowest value is then reached in fibrillation.

The presented methodology reveals a new perspective on the automatic detection of pro-arrhythmic regions in cardiac tissue, as the availability of cardiac data continues to improve^57^. With mapping of a sufficient spatial resolution, we can use tissue-averaged values of RQA measures, and their spatial correlation, to uncover the extent of spatial organisation of cardiac activity on the tissue scale. This proves to be a key property in distinguishing fibrillation driven by self-sustaining chaos, and that produced by an anchored re-entry exhibiting wavebreak. Limiting the problem to specifically distinguishing these two classes of arrhythmia, the spatial correlation of the RQA measure DET appears suitable up to a separation of measurement locations of 4 mm. The technology of high-resolution voltage mapping is advancing rapidly, with new innovations continuously being developed. For example, in a recent study^58^, the use of a new high-definition multi-electrode mapping catheter with inter-electrode spacing of just 3mm resulted in significant improvements in ventricular tachycardia ablation procedures. Additionally, another study^25^ used a high-resolution multi-electrode mapping catheter with inter-electrode spacing of only 2mm to accurately pinpoint areas of residual endocardial and epicardial conduction across myocardial infarcts, leading to successful myocardial infarction block with ablation. As these technologies continue to advance, we can expect further improvements in the accuracy and effectiveness of cardiac mapping.

A limitation of our study is that the membrane potential time series has been treated as available free of noise, whilst practical use of such an approach for arrhythmia classification would involve dealing with noisy signals. Noise spuriously reduces measures of predictability such as DET, and can hence compromise the differentiation of arrhythmia categories presented here. One compelling option to address this is to pre-process the membrane potential time series before attempting to calculate any RQA measures, and indeed automated noise removal that preserves action potentials has been recently demonstrated^59^. Options for making RQA robust to any residual noise after pre-processing are also available. Careful selection of the recurrence detection threshold, $\epsilon$, in equation (2) can reduce the influence of noise^60^, and noise-robust variants of some of the most useful RQA measures (in particular DET) are available^61^.

Our studies use a fixed ionic model, and treat tissue properties (such as conductivity) as homogeneous. This is a simplification, as cardiac tissue is naturally heterogeneous^62^, and variability in cell electrophysiological properties is well-established both spatially within a heart chamber^63^, and between members of a population^64^. RQA is appealing in that its metrics quantify fundamental properties such as the extent of order or periodicity evident in a time series, qualities that are expected to remain similar even if the AP changes shape (although changes in AP duration might affect periodicity measures). Additionally, our spatial analysis calculates RQA quantities separately for each individual location’s time series, and each calculation thus perceives only a single conductivity value. Given these observations we anticipate that the RQA-based approach we take here is relatively robust to variability in cell and tissue properties, although of course validating this intuition is an important direction for future research.

## Supplementary Information

Supplementary Information.

## Data Availability

The datasets used and/or analysed during the current study are available from the corresponding author on reasonable request.
