# Supplementary material for: Recurrence quantification analysis for fine-scale characterisation of arrhythmic patterns in cardiac tissue
Source: Sci Rep. 2023 Jul 22;13:11828. doi: 10.1038/s41598-023-38256-w (PMC10363137; doi:10.1038/s41598-023-38256-w)
Supplement: Supplementary file 1 — Supplementary Information. [file 41598_2023_38256_MOESM1_ESM.pdf]

Supplementary Materials

The Fenton–Karma Model

The Fenton–Karma model is a simplified model of cardiac cell electrophysiology that describes the net effects of transmembrane ion flow via only three currents — a fast inward current (J_{fi}, roughly equivalent to the fast Na^+ current), a slow inward current (J_{si}, roughly equivalent to the L-type Ca^{2+} current), and a slow outward current (J_{so}, roughly equivalent to the combined effect of outwards K^+ currents). A full justification of the model's form is provided in the work of its namesake authors (reference provided in main text). Here the model definition is provided for convenience, and the parameters used given in Table S1.

Given the Heaviside function,

$$H(x) = \begin{cases} 0 & x \leq 0 \\ 1 & x > 0 \end{cases},$$

the Fenton–Karma model takes the form

$$J_{\text{ion}} = -J_{\text{fi}} - J_{\text{so}} - J_{\text{si}}, \tag{1}$$

where the definitions of the three currents described above are

$$\begin{aligned} J_{fi} &= -H(u-u_c)\,\frac{v}{\tau_d}(1-u)(u-u_c) \\ J_{so} &= \big(1-H(u-u_c)\big)\frac{u}{\tau_o} + H(u-u_c)\frac{1}{\tau_r} \\ J_{si} &= -\frac{w}{\tau_{si}}\frac{1+\tanh\big(k(u-u_c^{si})\big)}{2}. \end{aligned}$$

Here v and w are gating variables, their values defined by ordinary differential equations that couple with equation (4) above,

$$\begin{aligned} \frac{dv}{dt} &= \big(1-H(u-u_c)\big)\frac{1-v}{\tau_v^-} - H(u-u_c)\frac{v}{\tau_v^+}, \qquad \tau_v^- = \big(1-H(u-u_v)\big)\tau_{v1}^- + H(u-u_v)\tau_{v2}^- \\ \frac{dw}{dt} &= \big(1-H(u-u_c)\big)\frac{1-w}{\tau_w^-} - H(u-u_c)\frac{w}{\tau_w^+}. \end{aligned}$$

Recall that u is a dimensionless version of the membrane potential, scaled such that $0 \leq u \leq 1$.

Table S1. The parameters used for the Fenton–Karmal model of cardiac cell electrophysiology.

Parameter	Value	Definition
C_m	1	Cell capacitance
τ_d	0.395 ms	Time constant defining fast inward current strength
τ_r	33.33 ms	Time constant defining slow outward current strength (activated cell)
τ_o	9 ms	Time constant defining slow outward current strength (inactivated cell)
τ_{si}	29 ms	Time constant defining slow inward current strength
τ_v^+	3.33 ms	Time constant for fast inward current inactivation
τ_{v1}^-	9 ms	First time constant for fast inward current recovery
τ_{v2}^-	8 ms	Second time constant for fast inward current recovery
τ_w^+	250 ms	Time constant for slow inward current inactivation
τ_w^-	60 ms	Time constant for slow inward current recovery
u_c	0.13	Threshold for u for fast inward activation
u_v	0.04	Threshold for u for switch between $\tau_{v_1}^-$ and $\tau_{v_2}^-$
u_c^{si}	0.5	Threshold for u for slow inward current activation
k	15	Constant defining steepness of u-dependence for slow inward current

Supplementary Data and Figures

Table S2. Values of each time series metric, averaged spatially over the tissue, for an example of each class of activation.

	**Single Rotor**		**Fibrillation**	
**Variable**	**Anchored**	**Travelling**	**Driven by rotor**	**Self-sustained**
RQA (REC)	0.027	0.006	0.004	0.004
RQA ($RATIO$)	38.606	189.934	260.985	264.490
RQA (DET)	0.996	0.993	0.992	0.993
RQA (DIV)	0.011	0.032	0.035	0.035
RQA (L_{max})	214.181	36.149	32.849	32.802
RQA (L_{mean})	19.362	17.729	16.882	17.372
RQA ($ENTR$)	3.440	2.559	2.506	2.477
RQA (LAM)	0.973	0.891	0.833	0.830
RQA (V_{max})	13.881	11.254	10.210	9.980
RQA (V_{mean})	6.597	4.214	3.929	3.821
$OI1$	0.387	0.194	0.251	0.231
$OI2$	0.475	0.317	0.364	0.361
$EIG1$	43.497	10.964	9.715	8.856
$EIG2$	41.660	9.604	8.659	7.978
$ApEnt$	0.043	-0.011	0.022	0.007
$SampEnt$	0.187	0.262	0.218	0.211

Table S3. Values of the spatial correlation (Moran's I) of each time series metric, for an example of each class of activation.

	**Single Rotor**		**Fibrillation**	
**Variable**	**Anchored**	**Travelling**	**Driven by rotor**	**Self-sustained**
RQA (REC)	0.100	0.735	0.412	0.824
RQA ($RATIO$)	0.627	0.884	0.900	0.873
RQA (DET)	0.616	0.416	0.508	0.250
RQA (DIV)	0.455	0.735	0.593	0.610
RQA (L_{max})	0.569	0.621	0.703	0.686
RQA (L_{mean})	0.598	0.711	0.622	0.623
RQA ($ENTR$)	0.763	0.616	0.644	0.577
RQA (LAM)	0.669	0.795	0.799	0.753
RQA (V_{max})	0.130	0.613	0.658	0.629
RQA (V_{mean})	0.163	0.712	0.711	0.686
$OI1$	0.798	0.933	0.840	0.901
$OI2$	0.805	0.944	0.847	0.891
$EIG1$	0.187	0.559	0.399	0.567
$EIG2$	0.605	0.562	0.665	0.602
$ApEnt$	0.682	0.741	0.524	0.569
$SampEnt$	0.729	0.773	0.650	0.610

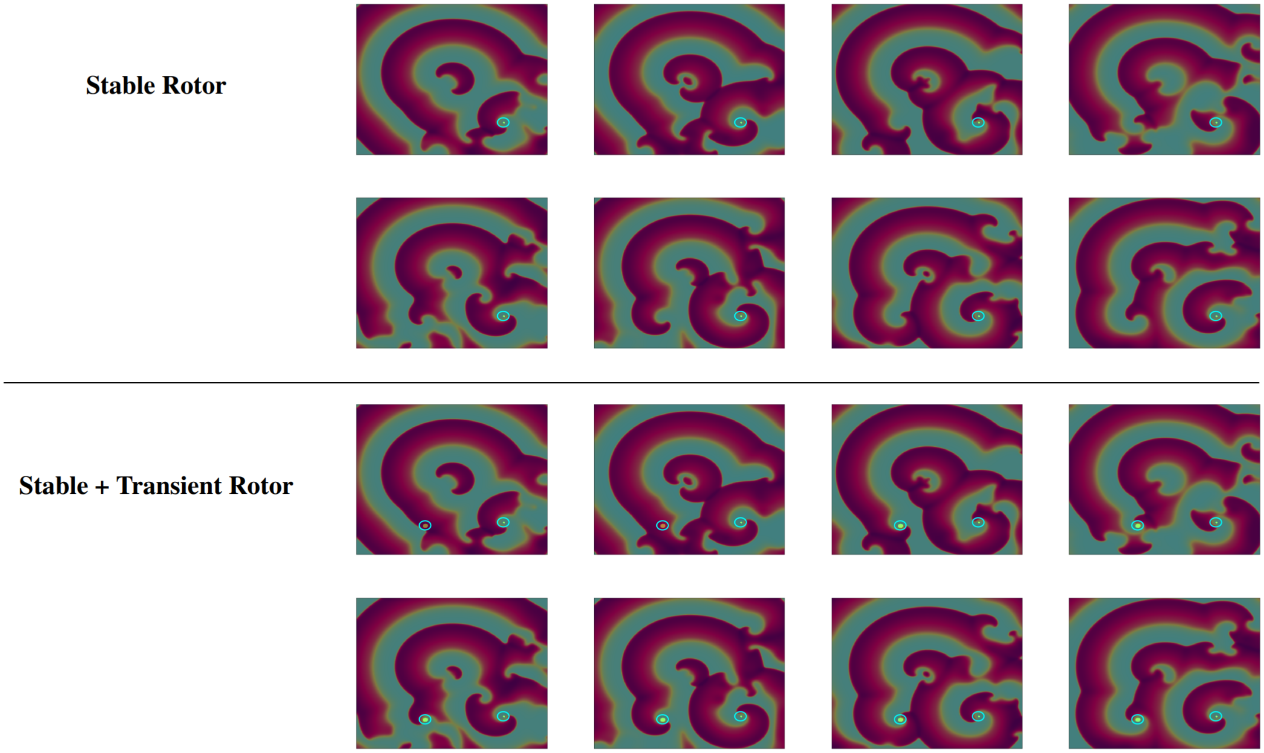

Figure S1. Detection of temporarily localized rotors via RQA measures. Choosing the threshold $L_{mean} \geq 30$, varying the threshold for L_{max} allows some selection of how long a rotor must persist in one location to be detected. Choosing $L_{max} \geq 800$, only the rotor that persists during the whole time window is detected (small highlighted region within the light blue circle, top two rows). Reducing this threshold to $L_{max} \geq 300$, the rotor that eventually forms in the bottom left and then persists for the remainder of the time window is also detected (additional highlighted region within the light blue circle, bottom two rows).

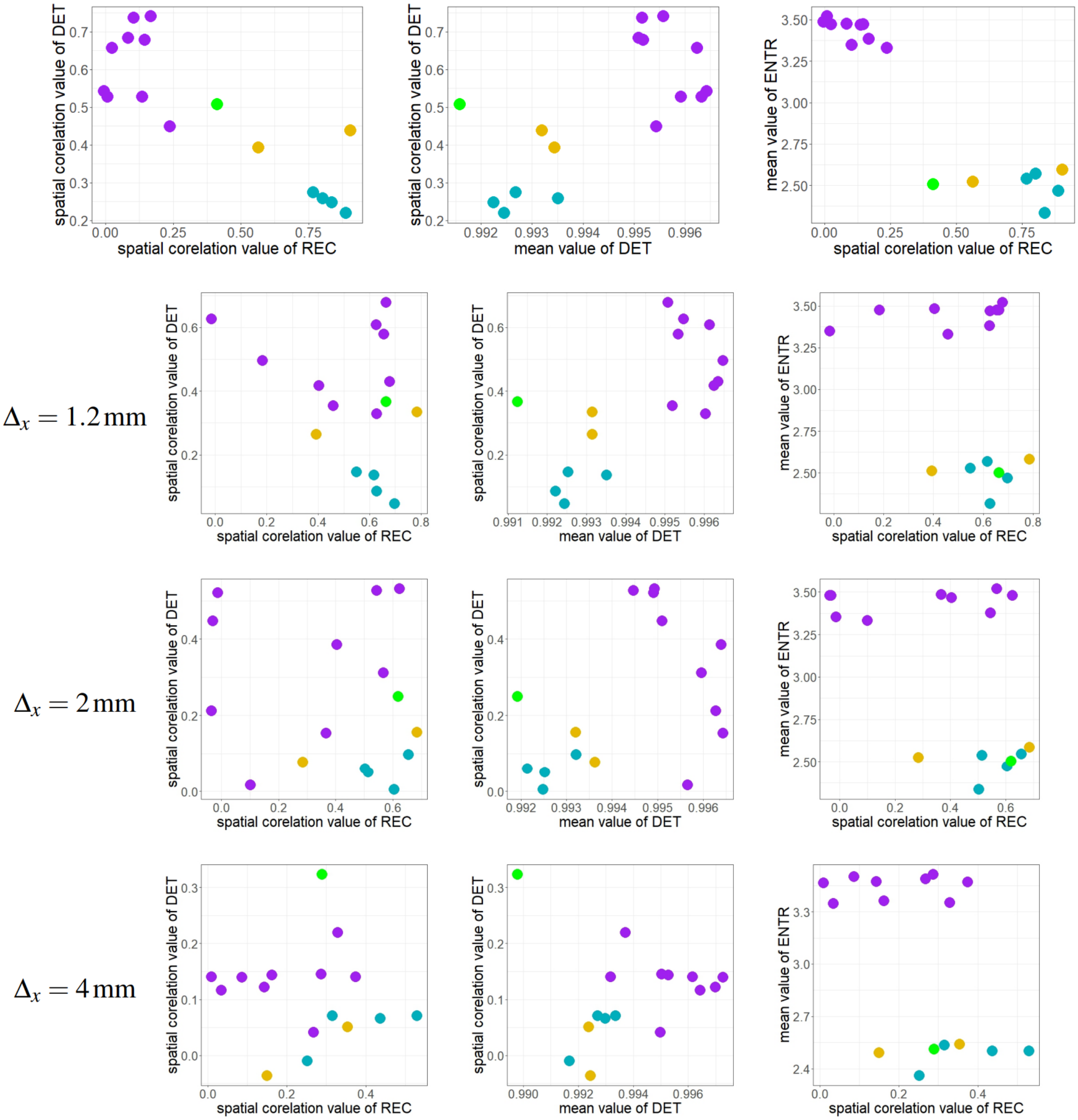

Figure S2. Scatter plots of selected pairs spatial metrics (columns) calculated using different spatial sampling resolutions (rows), for different classifications of arrhythmia (fibrillation-blue; anchored rotation-purple; fibrillation with anchored rotation-green; traveling rotor-orange). When the time series used for metric calculation are taken from sites with greater spatial separation, the ability to distinguish the different arrhythmia classifications generally degrades, however some classifications appear separable from the others for even the largest Δ_x value, via mean ENTR and spatial correlation of DET.
